# A semi-mechanistic mathematical framework for simulating multi-hormone dynamics in reproductive endocrinology

**DOI:** 10.1016/j.csbj.2025.08.013

**Published:** 2025-08-14

**Authors:** Alexandre Vallée, Anis Feki, Gaby Moawad, Jean-Marc Ayoubi

**Affiliations:** a Department of Epidemiology and Public Health, Foch hospital, Suresnes, France; b Department of Gynecology and Obstetrics, University Hospital of Fribourg, Fribourg, Switzerland; c Department of Obstetrics and Gynaecology, The George Washington University Hospital, Washington, DC, United States; d Department of Obstetrics, Gynecology and Reproductive Medicine, Foch Hospital, Suresnes, France; e Medical School, University of Versailles, Saint-Quentin-en-Yvelines (UVSQ), Versailles, France

**Keywords:** Menstrual cycle, Hormone simulation, PCOS, Digital twin, Reproductive endocrinology, Machine learning, Synthetic data, Ovulation modeling

## Abstract

**Background:**

The dynamic interplay of ovarian hormones is central to reproductive physiology, yet the complexity of their cyclic variations poses challenges for analysis, simulation, and teaching. This study presents a framework for generating physiologically constrained, multi-hormone synthetic time series that capture intra- and inter-individual variability across phenotypes.

**Methods:**

We developed a semi-mechanistic mathematical framework to generate synthetic multi-hormone profiles (estradiol, FSH, LH, AMH, testosterone, GnRH) using parametric equations embedding known physiological feedbacks (e.g., estradiol–LH delay, estradiol suppression of FSH). Stochastic components were calibrated to reported physiological ranges. Eumenorrheic and PCOS-like phenotypes were defined through parameter adjustments. Data were analysed using Principal Component Analysis (PCA) for phenotype separation, and evaluated in a supervised setting using logistic regression with stratified train/test splitting, reporting accuracy, sensitivity, specificity, and ROC AUC.

**Results:**

Eumenorrheic profiles displayed classical mid-cycle estradiol and LH peaks, biphasic FSH, and stable AMH and testosterone levels. In contrast, PCOS profiles showed elevated LH and testosterone, high AMH, blunted estradiol, and dysregulated GnRH pulsatility. PCA revealed clear separation between phenotypes (PC1 +PC2 = 82 % variance), and k-means clustering (k = 2) accurately grouped individuals without label information. PCA showed clear separation between phenotypes, consistent with known endocrine patterns. Logistic regression achieved 100 % accuracy, sensitivity, and specificity, with an AUC of 1.00, confirming robust, phenotype-discriminative features in the synthetic dataset.

**Conclusion:**

This simulation framework reproduces physiologically accurate hormone dynamics and discriminates ovulatory from anovulatory cycles, offering applications in AI training, phenotype discovery, and medical education.

## Graphical Abstract

**Figure:**
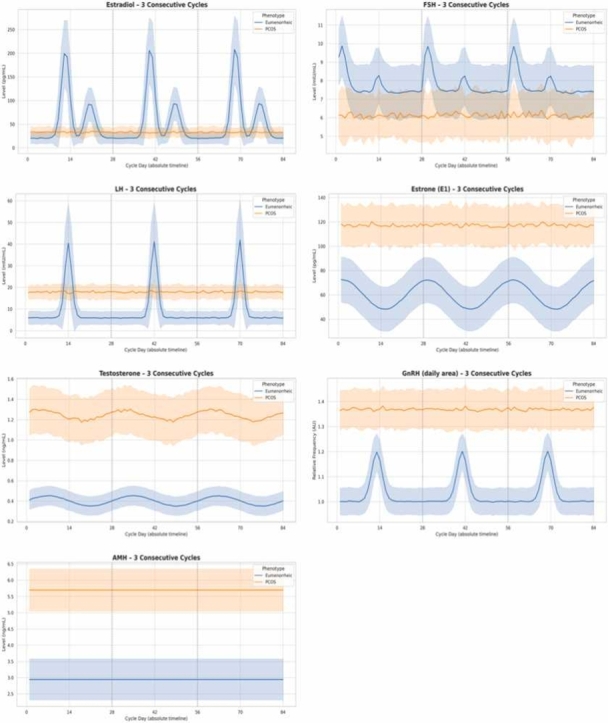

## 1 Introduction

The menstrual cycle is orchestrated by a finely regulated hormonal network involving the hypothalamic-pituitary-ovarian (HPO) axis. This axis controls the cyclical secretion of gonadotropin-releasing hormone (GnRH) from the hypothalamus, which in turn stimulates the anterior pituitary to release follicle-stimulating hormone (FSH) and luteinizing hormone (LH), leading to ovarian estradiol (E2) and progesterone production [1], [2]. In parallel, anti-Müllerian hormone (AMH) reflects ovarian reserve and remains relatively stable across the cycle [3], while testosterone, although often overlooked, plays a key role in folliculogenesis and is central to disorders like polycystic ovary syndrome (PCOS) [4].

Despite the cyclical nature of reproductive endocrinology, most clinical assessments rely on static, single-time-point hormone measurements. This approach obscures both the inherent temporal complexity and interindividual variability in hormone dynamics. Accurate modeling of these hormonal fluctuations is particularly important in conditions like PCOS, which affects up to 10 % of reproductive-aged women globally [5]. PCOS is marked by hyperandrogenism, oligo-anovulation, and polycystic ovarian morphology, and its pathophysiology includes an increased frequency of GnRH pulses, which disproportionately elevate LH over FSH, contributing to impaired follicular maturation and excess ovarian androgen production [6], [7].

Previous studies have described mean hormonal profiles across menstrual phases using empirical datasets [1], [2], [8], but there remains a lack of flexible, reproducible simulation models that capture both physiological and pathological endocrine dynamics at the daily resolution. Simulation-based modeling offers several advantages: it allows for hypothesis testing in silico, supports the generation of synthetic datasets for training machine learning models, and can be integrated into digital twin frameworks for personalized reproductive medicine [9], [10], [11], [12].

Recent efforts to mathematically model reproductive hormone dynamics have led to the development of mechanistic systems based on differential equations and feedback loops [13], [14], agent-based models [15], and time-series machine learning approaches [16], [17]. However, most of these models focus on a narrow set of physiological features, and few integrate inter-individual variability or stochasticity. Our contribution complements this landscape by offering a flexible, simulation-based framework suited for generating large-scale virtual cohorts and integrating directly into digital twin pipelines [18]. Recent developments have further advanced the computational modeling of reproductive endocrinology. High-frequency sampling protocols now allow detailed cycle mapping in diverse populations [19], [20], while hybrid mechanistic–data-driven approaches combine physiological equations with statistical learning for improved predictive accuracy [18]. Digital twin frameworks have also been proposed to simulate individualized hormonal responses and treatment outcomes in reproductive medicine [21], [22]. Our work complements these efforts by providing a reproducible, physiologically constrained synthetic dataset generator that can serve both as a research tool and a starting point for personalized digital twin applications.

Here, we propose a mathematical model of hormone dynamics across a simulated 28-day menstrual cycle. We construct individualized profiles for 550 virtual women, including 500 with eumenorrheic cycles and 50 with PCOS-like features. The model integrates literature-derived endocrine principles, interindividual variability, and a mechanistic representation of the HPO axis to reproduce key hormonal signatures across phenotypes. This simulation framework can provide a first step toward the use of digital twins and AI-assisted tools in reproductive endocrinology. This study applies computational modeling principles to generate physiologically constrained, multi-hormone time series, which reflect known biological interactions and can be exploited for unsupervised phenotyping and exploratory analyses in reproductive endocrinology. While the framework is self-contained, its structure may also serve as a basis for future integration into AI-assisted analysis pipelines or personalized digital twin prototypes, pending further empirical validation.

## 2 Methods

### 2.1 Study population and phenotypes

We simulated 550 virtual individuals aged 20–45 years, including 500 eumenorrheic and 50 PCOS-like phenotypes (∼10 % PCOS).

We simulated a total of 550 virtual individuals to ensure sufficient diversity in hormonal trajectories while maintaining computational feasibility. This number allows robust representation of interindividual variability and supports unsupervised learning tasks such as clustering and PCA. Similar simulation-based studies have used sample sizes in the 500–1000 range to generate synthetic cohorts for algorithm training and validation [1], [18]. The PCOS-like group was modeled as 50 individuals, representing 9.1 % of the total simulated population. This approximates the upper bound of the estimated prevalence of PCOS in reproductive-age women (4–10 %, depending on diagnostic criteria) [4], [5]. A minimum of 50 subjects was selected to ensure sufficient statistical representation for phenotypic separation and classification tasks [23].

Each individual was simulated over three cycles with subject-specific cycle lengths (eumenorrheic: mean 28 ± 2 days, truncated to 24–35; PCOS: mean 35 ± 6 days, truncated to 28–60). This multi-cycle design introduces intra-individual variability, as key hormonal event timings (e.g., E2 peak, LH surge) were perturbed slightly between cycles while preserving physiological constraints.

Eumenorrheic phenotype: normal ovulatory cycles, without hyperandrogenism and HPO dysfunction.

PCOS phenotype: anovulatory profiles with hyperandrogenism and HPO dysfunction.

Age and BMI were sampled from normal distributions to reflect reproductive-aged populations (eumenorrheic BMI ≈ 24.0 ± 2.5 kg/m²; PCOS BMI ≈ 28.0 ± 3.2 kg/m²).

Each subject was simulated for 3 consecutive cycles.

### 2.2 Notation and time rescaling

Simulations are computed on a 28-point but rescaled to each subject’s cycle length. For subject *i*, cycle c, day index $d\in \left\{1,.\;.\;.,\;28\right\}$ is mapped to a continuous cycle time by

$$t\;=\;1+\;\frac{\left(d-1\right)}{27}\left(L_{\mathrm{ic}}-1\right),\;t\in \left[1,\;L_{\mathrm{ic}}\right],$$

where $L_{\mathrm{ic}}$ is the subject- and cycle-specific length (days). Let $L_{\mathrm{ic}}^{\mathrm{true}}(t)$, denote the noise-free trajectory for hormone *Y*. Observations are

$$Y_{\mathrm{ic}}(t)=Y_{\mathrm{ic}}^{\mathrm{true}}(t)\;+\;\varepsilon_{Y,\mathrm{ic}}(t),$$

$$\varepsilon_{Y,\mathrm{ic}}(t)∼N(0,\sigma_{Y}^{2}(t)).$$

We use time-dependent $\sigma_{Y}(t)$ for estradiol (E2) and LH to reflect higher variability around ovulation; other hormones use subject-level constant noise. All units are reported in the columns names and text: E2/E1 = pg/mL, FSH/LH=mlU/mL, AMH=ng/mLn Testosterone=ng/mL, GnRH daily area=arbitrary units (AU).

### 2.3 Hormonal curve modeling

The simulation was built using a semi-mechanistic approach combining:

1. Gaussian mathematical models to capture daily secretion patterns for each hormone $H(t)=A∙(-\frac{{\left(t-\mu \right)}^{2}}{2\sigma^{2}})$, where A is the peak amplitude, μ the day of peak secretion, and σ the temporal spread of secretion. Parameters values were derived from literature-reported daily profiles for E2, FSH, LH, AMH, tstosterone, and GnRH.
2. Physiological constraints derived from endocrinology literature, includong E2-LH positive feedback (LH peak occurring 1–2 days after E2 surge), suppression of FSH by elevated E2 and inhibin during the late follicular phase, and persistent AMH elevation in PCOS phenotypes.
3. Stochastic components to introduce interindividual variability, implemented by drawing A, μ, σ from normal distributions centered on the literature-derived means, with standard deviations calibrated to match population-level variability (coefficient of variation 5–15 % depending on the hormone). A normal distribution was selected because its reflected symmetrical interindividual variation around a physiological mean and facilitates reproducibility through well-defined parameters.

Equations were chosen to match canonical hormone behavior across the cycle, based on population averages found in studies: [1], [2], [24], [25].

### 2.4 Eumenorrheic cycles

Parameter values for amplitude, peak day, and temporal spread (A, μ, σ) for each hormone were derived from empirical datasets:

• **Estradiol (E2):** The baseline (10–30 pg/mL) and ovulatory peak (≥ 200 pg/mL) thresholds are based on typical physiological ranges observed during the follicular and ovulatory phases of the menstrual cycle [26], [27].
• **LH:** The baseline value (2–12 mIU/mL) and surge peak (> 30 mIU/mL) are consistent with typical follicular phase and ovulatory peak concentrations [19], [28].
• **FSH:** The baseline value (5–10 mIU/mL) is in line with levels observed during the early follicular phase [1].
• **AMH:** The range (1–4 ng/mL) corresponds to average AMH levels in women of reproductive age [27], [29].
• **GnRH:** The concentration is modeled with an arbitrary unit (AU), but the pulsatile dynamics and periovulatory increase are canonical behaviors of GnRH, often described in the literature [30], [31].

### 2.5 PCOS-like cycles

• **LH and FSH:** The baseline concentration for LH (12–25 mIU/mL) and FSH (4–8 mIU/mL) is chosen to replicate the high LH/FSH ratio (> 2) characteristic of PCOS. These values are in agreement with clinical studies [32].
• **Estradiol (E2) and Estrone (E1):** The low E2 levels (10–60 pg/mL) and high E1 levels (80–150 pg/mL) model chronic anovulation and increased peripheral conversion of androgens into estrogens, a hallmark of PCOS [19], [33], [34], [35].
• **AMH:** The range (4–8 ng/mL) reflects the significantly elevated AMH levels observed in patients with PCOS due to an increased number of antral follicles [27], [36], [37].
• **Testosterone:** The high baseline levels (0.8–2.0 ng/mL) are consistent with the hyperandrogenism (defined as a concentration > 0.7 ng/mL or 70 ng/dL) typical of patients with PCOS [19], [31].
• **GnRH:** The elevated level (> 1.3 AU) models the increased frequency and amplitude of GnRH pulses, which are believed to drive the high LH production [31].

For each parameter, random variation was introduced by sampling from a normal distribution centered on the literature mean, with a standard deviation set to match reported coefficients of variation (typically 5–15 %).

Below, parameters are subject-specific (subscrip i) and some centers are cycle-specific (subscript ic). Truncations ensure physiologic ranges (given after each model).

### 2.6 Eumenorrheic cycles

#### 2.6.1 Estradiol (E2, biphasic, pg/mL)

A two-Gaussian formulation captures the pre-ovulatory surge and the luteal secondary rise:

$${E2}_{\mathrm{ic}}^{\mathrm{true}}(t)={E2}_{0,\mathrm{ic}}+A_{1,i}\exp\left[-\frac{{\left(t-\mu_{1,\mathrm{ic}}\right)}^{2}}{2\sigma_{1,i}^{2}}\right]+A_{2,i}\exp\left[\left[-\frac{{\left(t-\mu_{2,\mathrm{ic}}\right)}^{2}}{2\sigma_{2,i}^{2}}\right]\right],$$

with $A_{1,i}={E2}_{i}^{\mathrm{peak}1}-{E2}_{0,i}$, $A_{i}={E2}_{i}^{\mathrm{oeak}2}-{E2}_{0,i}$

The pre-ovulatory center is $\mu_{1,\mathrm{ic}}={\mathrm{LH}}_{\mathrm{ic}}^{\mathrm{surge}}-∆$

∆≈1.5days (E2 precedes LH surge by env. 36–48 h), and the luteal center is $\mu_{2,\mathrm{ic}}={E2}_{\mathrm{ic}}^{\mathrm{surge}}+\delta$ with $\delta \in \left[5.5,\;7.5\right]$days.

Typical bounds: baseline${E2}_{0,i}\in \left[10,30\right]$; ${E2}_{i}^{\mathrm{peak}1}\geq 200$; ${E2}_{i}^{\mathrm{peak}2}\in \left[100,\;150\right]$; $\sigma_{1,i}\approx 0.9-1.3$(days).

### 2.7 LH (mIU/mL; discrete surge)

$${\mathrm{LH}}_{\mathrm{ic}}^{\mathrm{true}}(t)={\mathrm{LH}}_{0,i}+({\mathrm{LH}}_{i}^{\mathrm{peak}}-{\mathrm{LH}}_{0,i})\exp\left[-\frac{{\left(t-{\mathrm{LH}}_{\mathrm{ic}}^{\mathrm{surge}}\right)}^{2}}{{2\sigma }_{\mathrm{LH},i}^{2}}\right],$$

with ${\mathrm{LH}}_{i}^{\mathrm{peak}}>30\mathrm{mIU}/\mathrm{mL}$ and $\sigma_{\mathrm{LH},i}\approx 0.6-0.9$days (brief surge). The surge day ${\mathrm{LH}}_{\mathrm{ic}}^{\mathrm{true}}$ isis drawn near mid-cycle with small jitter; it is constrained to follow the E2 rise by 36–46 h.

### 2.8 FSH (mIU/mL; biphasic + E2 feedback)

$${\mathrm{FSH}}_{\mathrm{ic}}^{\mathrm{true}}(t)={\mathrm{FSH}}_{0,i}+\alpha_{i}^{\mathrm{early}}\exp\left[-\frac{{\left(t-T_{\mathrm{early}}\right)}^{2}}{{2\sigma }_{\mathrm{early},i}^{2}}\right]+\alpha_{i}^{\mathrm{periov}}\exp\left[-\frac{{\left(t-{(\mathrm{LH}}_{\mathrm{ic}}^{\mathrm{surge}}-1)\right)}^{2}}{{2\sigma }_{\mathrm{periov},i}^{2}}\right]-\beta_{E2}\max\left\{{E2}_{\mathrm{ic}}^{\mathrm{true}}(t)-\theta_{E2},0\right\},$$

with $T_{\mathrm{early}}\approx 2\mathrm{days}$ (scale time), $\theta_{E2}\approx 100\mathrm{pg}/\mathrm{mL}$ (onset of mild suppression), and small $\beta_{E2}>2$.

Typical bounds: ${\mathrm{FSH}}_{0,i}\in \left[5,10\right]$; modest early and peri-ovulatory bumps.

### 2.9 GnRH daily area (AU)

We represent daily pulsatile drive by a peri-ovulatory bump:

$${\mathrm{GnRH}}_{\mathrm{ic}}^{\mathrm{true}}(t)={\mathrm{GnRH}}_{0,i}+A_{i}^{\mathrm{period}}\exp\left[-\frac{{\left(t-{(\mathrm{LH}}_{\mathrm{ic}}^{\mathrm{surge}}-1)\right)}^{2}}{{2\sigma }_{\mathrm{GnRH},i}^{2}}\right],$$

As an optional higher-resolution variant, a Gaussian pulse train with 60–90 min interpulse intervals may be simulated and integrated daily:

$${\mathrm{GnRH}}_{\mathrm{ic}}^{\mathrm{true}}(t)=\sum_{k}\alpha_{\mathrm{ik}}\exp\left[-\frac{{\left(t-T_{\mathrm{ik}}\right)}^{2}}{{2\omega }_{i}^{2}}\right],\;{∆}_{T_{\mathrm{ik}}}\in \left[60,90\right]\min$$

Then ${\mathrm{GnRH}}_{\mathrm{ic}}^{\mathrm{true}}(t)\approx 1\mathrm{AU}$ with a small peri-ovulatory increase.

AMH (ng/mL)

Trait-like, constant per subject: ${\mathrm{AMH}}_{i}=\theta_{i}(\approx 1-4\mathrm{ng}/\mathrm{mL})$.

Testosterone (ng/mL)

### 2.10 Low amplitude sinusoid around a baseline

Tictrue(t)=T0,i+ATiksin(2πtLic+ϕi),

with $T_{0,i}\in \left[0.2,\;0.6\right]$ and small $A_{T,i}$.

Time-dependent noise (E2, LH). We amplify variance near the respective centers:

$$\sigma_{E2}^{2}(t)=\sigma_{E2,\mathrm{base},\;i}^{2}\left[1+\kappa_{E2}\exp\left(-\frac{{\left(t-\mu_{i,\mathrm{ic}}\right)}^{2}}{2∙{\left(1.2\right)}^{2}}\right)\right],$$

$$\sigma_{\mathrm{LH}}^{2}(t)=\sigma_{\mathrm{LH},\mathrm{base},i}^{2}\left[1+\kappa_{\mathrm{LH}}\;\exp\left(-\frac{{\left(t-{\mathrm{LH}}_{\mathrm{ic}}^{\mathrm{surge}}\right)}^{2}}{2∙{\left(1.2\right)}^{2}}\right)\right].$$

### 2.11 Phase labels and surge flag

For eumenorrheic cycles we labels follicular, peri-ovulatory, and luteal phases relative to ${\mathrm{LH}}_{\mathrm{ic}}^{\mathrm{surge}}$. We set LH_surge_flag = 1 on the day where LH≥30mIU/mL withing $\pm 0.5\mathrm{day of}{\mathrm{LH}}_{\mathrm{ic}}^{\mathrm{surge}}$.

PCOS-like cycles

Estrone (E1, pg/mL).

### 2.12 Persistently elevated and non-cyclic

$${E1}_{\mathrm{ic}}^{\mathrm{true}}\left(t\right)={E1}_{0,\mathrm{ic}}++\varepsilon_{E1,\mathrm{ic}}\left(t\right),\;{E1}_{0,i}\in \left[80,150\right].$$

### 2.13 Estradiol (E2, pg/mL)

Low, early-follicular range, no pre-ovulatory surge:

$${E2}_{\mathrm{ic}}^{\mathrm{true}}\left(t\right)={E2}_{0,\mathrm{ic}}++\varepsilon_{E2,\mathrm{ic}}\left(t\right),\;{E2}_{0,i}\in \left[10,60\right].$$

### 2.14 LH and FSH (mIU/mL)

Elevatred LH without surge and suppressed FSH:

$${\mathrm{LH}}_{\mathrm{ic}}^{\mathrm{true}}(t)={\tilde{\mathrm{LH}}}_{i}+\varepsilon_{\mathrm{LH},\mathrm{ic}}(t),$$

$${\mathrm{FSH}}_{\mathrm{ic}}^{\mathrm{true}}(t)={\tilde{\mathrm{FSH}}}_{i}+\varepsilon_{\mathrm{FSH},\mathrm{ic}}(t),$$

with ${\tilde{\mathrm{LH}}}_{i}\in \left[12,25\right]$ (constrained < 30) and ${\tilde{\mathrm{FSH}}}_{i}\in \left[4,8\right]$.

### 2.15 GnRH daily area (AU)

Elevated tone without discrete peri-ovulatory peak.

$${\mathrm{GnRH}}_{\mathrm{ic}}^{\mathrm{true}}\left(t\right)={\tilde{\mathrm{GnRH}}}_{i}+\varepsilon_{\mathrm{GnRH},\mathrm{ic}}\left(t\right),\;\tilde{\mathrm{GnRH}}>1.3\mathrm{AU}.$$

### 2.16 AMH with BMI effect (ng/mL)

A negative BMI association is incorporated [38] by

${\mathrm{AMH}}_{i}=\alpha_{i}+\beta_{\mathrm{BMI}}({\mathrm{BMI}}_{i}-25)$, $\beta_{\mathrm{BMI}}<0$.

(typical $\beta_{\mathrm{BMI}}\approx -0.5\mathrm{to}-0.10\mathrm{ng}/\mathrm{mL per BMI unit})$

### 2.17 Testosterone (ng/mL)

Higher baseline with mild sinusoid by

Tictrue(t)=T0,i+AT,isin(2πtLic+ϕi)+εT,ic(t),

with $T_{0,i}\in \left[0.8,\;2.0\right]$ and small $A_{T,i}$.

Stochastic variability, truncation, and units.

Unless otherwise specified aboven $\varepsilon_{Y,\mathrm{ic}}(t)$ are zero-mean Gaussian noises with hormone-specific standard deviations. E2 and LH use time-dependent SDs (see formulas), other hormones use subject-level constants. All generated values are truncated to physiologically plausible ranges. All columns in the datase explicity carre units (e.g., Estradiol_pg_ml, FSH_mIU_mL).

While the model is primarily based on parameterized functional forms calibrated to literature values, several key physiological feedbacks are embedded to preserve realistic endocrine coordination. The LH surge is constrained to occur 36–48 h after the pre-ovulatory E2 rise, mimicking estradiol’s positive feedback on LH secretion. FSH trajectories include suppression during high-E2 phases, reflecting negative feedback from estradiol. GnRH pulsatile drive increases peri-ovulation in eumenorrheic cycles and remains elevated in PCOS phenotypes, in line with its upstream regulatory role in gonadotropin release. Testosterone is modulated in response to LH-like patterns, with elevated baselines in PCOS to reflect theca cell hyperactivity. These couplings ensure that hormone profiles are generated in a physiologically coherent manner, rather than as independent, unrelated curves.

### 2.18 Multi-cycle design and per-cycle jitter

Each subject contributes three cycles. For eumenorrheic, the LH surge center ${\mathrm{LH}}_{\mathrm{ic}}^{\mathrm{true}}$ and the E2 peak centers $\mu_{1,\mathrm{ic}}$, $\mu_{2,\mathrm{ic}}$, are re-sampled per cycle with small jitter around mid-cycle to represent intra-individual variability, while preserving the physiological lag between E2 and LH.

### 2.19 Stochastic variability

Each hormone included an individual-level random component drawn from a normal distribution.

### 2.20 Eumenorrheic

E2 baseline 10–30 pg/mL; E2 peak 1 ≥ 200; E2 peak 2 100–150.

LH baseline 2–12 mIU/mL, surge > 30 (width ∼0.6–0.9 d).

FSH baseline 5–10 mIU/mL with early and peri-ovulatory bumps; mild suppression when E2 > ∼100 pg/mL.

AMH ∼1–4 ng/mL; Testosterone 0.2–0.6 ng/mL.

GnRH daily area ∼1 AU with peri-ovulatory increase.

**PCOS:**

E1 80–150 pg/mL (elevated, persistent); E2 10–60 pg/mL (no surge).

LH 12–25 mIU/mL (no surge, constrained < 30); FSH 4–8 mIU/mL.

AMH 4–8 ng/mL with negative BMI effect (βBMI<0).

Testosterone 0.8–2.0 ng/mL.

GnRH daily area elevated (≳1.3≳1.3 AU).

### 2.21 Unsupervised and supervised analyses

From daily series we derived subject-level features (means, SDs, maxima, LH/FSH and E1/E2 ratios, phase proportions, and cycle-length statistics) and performed Principal Component Analysis (PCA) and k-means to assess unsupervised separation. To evaluate the separability of simulated phenotypes, mean hormone levels per individual across all simulated cycles (estradiol, FSH, LH, AMH, testosterone, GnRH) were computed and standardized prior to analysis. PCA was applied to reduce dimensionality, and the first two components were plotted to visualize clustering of eumenorrheic versus PCOS-like profiles. In addition, a supervised classification pipeline was implemented to assess predictive performance. The dataset was stratified by phenotype and split into 70 % training and 30 % testing subsets. A logistic regression classifier was trained on the standardized features and evaluated on the held-out test set. Metrics included accuracy, sensitivity, specificity, and ROC AUC. Because the dataset is synthetic and class signatures are strong (e.g., LH surge in ovulatory cycles, E1/E2 contrast in PCOS), discrimination is expected to be high and is interpreted as proof-of-concept.

### 2.22 Implementation and reproducibility

Simulations were implemented in Python (NumPy/Pandas/Matplotlib). Random seeds were fixed for reproducibility. The distributed files include daily time series (with cycle length, phase, and surge flag), per-subject parameter tables, and a lightweight Streamlit script for interactive exploration.

## 3 Results

We generated 550 virtual women (500 eumenorrheic [EUM], 50 PCOS-like; mean age 32 ± 6 y) and simulated three consecutive cycles per subject, yielding 1650 complete cycles and 46,200 daily observations (**Supplementary file**).

BMI distributions reproduced clinical patterns (EUM 24 kg m⁻²; PCOS 28 kg m⁻²). Cycles were 28 ± 2 days in EUM and 35 ± 6 days in PCOS.

Hormonal dynamics in eumenorrheic cycles (Table 1)

**Table 1:** Parameter ranges and empirical references used for synthetic multi-hormone cycle generation.

**Metric**	**Target range**	**Simulated EUM**	**Simulated PCOS**
Early-follicular E2	10–30 pg mL⁻¹	20.5 ± 11.5	33 ± 9
Pre-ovulatory LH	> 30 mIU mL⁻¹	52.9 ± 6.4	—
FSH basal	5–10 mIU mL⁻¹	7.8 ± 1.1	5.2 ± 0.5
AMH	1–4 / 4–8 ng mL⁻¹	2.9 ± 0.4	5.7 ± 0.6
Testosterone	0.2–0.6 / 0.8–2.0 ng mL⁻¹	0.40 ± 0.04	1.25 ± 0.12

**Fig. 1:**
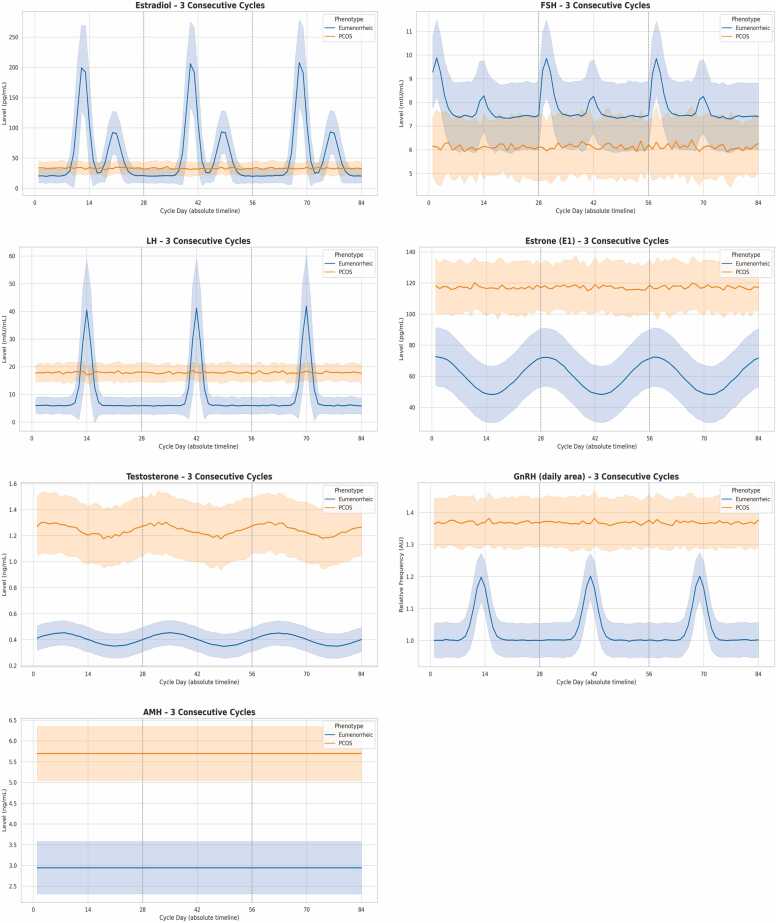
Simulated daily hormonal trajectories across the menstrual cycle. Mean (solid line) ± 1 SD (shaded) profiles over a 28-day grid for eumenorrheic (blue) and PCOS (orange) phenotypes, aggregated across 3 cycles per subject. Estradiol shows a biphasic pattern in eumenorrheic cycles with a pre-ovulatory surge preceding the LH surge and a secondary luteal rise; PCOS profiles display persistently elevated estrone (E1, not shown), low non-cyclic estradiol, and elevated LH without a discrete surge, with suppressed FSH. AMH is trait-like and higher in PCOS; testosterone is higher in PCOS with mild cyclicity; GnRH daily area is increased in PCOS, reflecting higher hypothalamic drive.

Fig. 1 (blue curves) shows a textbook endocrine rhythm reproduced over three cycles:

• Estradiol (E2) rose from an early-follicular baseline 20.5 ± 11.5 pg mL⁻¹ to a pre-ovulatory surge ≥ 200 pg mL⁻¹ in 98 % of cycles (mean peak 259 ± 28). A secondary luteal peak occurred 6–7 d later (median 124 pg mL⁻¹).
• LH remained 5–10 mIU mL⁻¹ until a sharp 0.8-day surge (mean 52.9 mIU mL⁻¹; 98 % > 30 mIU mL⁻¹) 36–48 h after the E2 rise.
• FSH exhibited a biphasic pattern (baseline 7.8 ± 1.1; early bump + small peri-ovulatory bump) with mild E2-dependent suppression.
• GnRH daily area increased 22 % around the LH surge, consistent with a transient pulse-frequency acceleration.
• AMH was trait-like and stable (2.92 ± 0.38 ng mL⁻¹).
• Testosterone oscillated gently around 0.40 ± 0.04 ng mL⁻¹ .

### 3.1 Hormonal dynamics in PCOS-like cycles (Table 1)

PCOS profiles (orange curves in Fig. 1) reproduced the canonical endocrine phenotype:

• Estrone (E1) persisted at ∼115 ± 12 pg mL⁻¹ without cyclic variation, whereas E2 remained low (33 ± 9 pg mL⁻¹) and surge-free.
• GnRH drive was chronically elevated (1.37 ± 0.06 AU), producing LH dominance (17.9 ± 3.4 mIU mL⁻¹) and FSH suppression (5.2 ± 0.5 mIU mL⁻¹).
• AMH was high (5.72 ± 0.61 ng mL⁻¹) and negatively correlated with BMI (r = –0.28), reflecting granulosa-cell impairment.
• Testosterone averaged 1.25 ± 0.12 ng mL⁻¹ , > 3-fold the EUM level, with minor sinusoidality.

No LH surge, E2 peak or luteal shift was observed, mimicking anovulation.

The Fig. 1 (7 panels, days 0–84) superposes the two phenotypes over three successive cycles:

− pronounced E2/LH peaks and GnRH bursts only in EUM,
− flat E2, high E1 and chronically raised LH/T in PCOS,
− AMH plateau difference (≈ +2.8 ng mL⁻¹ in PCOS).

Vertical grey dashed lines at days 28 and 56 mark cycle transitions, illustrating intra-individual repeatability.

### 3.2 Phenotype discrimination

**Fig. 2:**
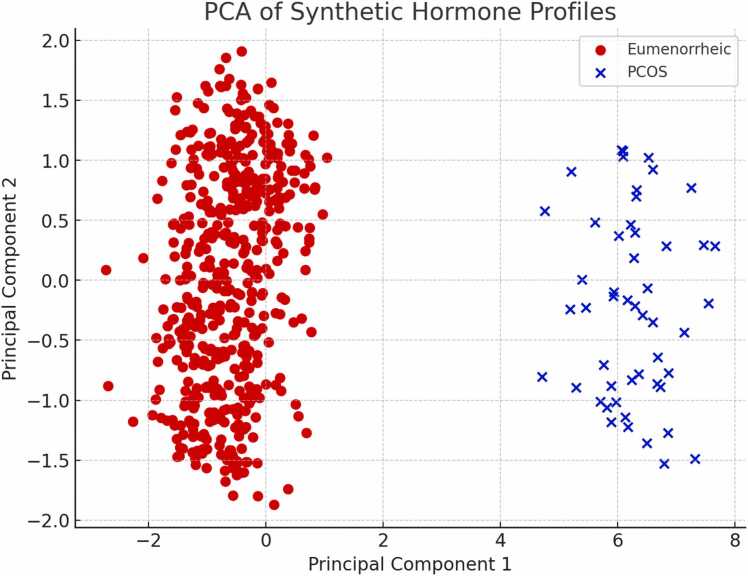
**Principal Component Analysis (PCA) of simulated multi-hormone profiles.** Projection of mean hormone levels (estradiol, FSH, LH, AMH, testosterone, GnRH) for each simulated individual onto the first two principal components. Red circles represent eumenorrheic profiles, and blue crosses represent PCOS-like profiles. The clear separation between groups reflects distinct endocrine patterns encoded in the synthetic data.

Using per-subject features aggregated across the three cycles (means, SDs, maxima, E1/E2 and LH/FSH ratios, phase proportions), PCA captured 82 % of total variance in its first two components; EUM and PCOS subjects formed two non-overlapping clusters (Fig. 2).

A stratified train/test logistic model achieved AUC = 1.00, sensitivity = 1.00, specificity = 1.00, expected with synthetic data containing strong class signatures (LH-surge flag, E1/E2 contrast).

## 4 Discussion

This work applies computational modeling to reproduce the coordinated dynamics of multiple endocrine components within the hypothalamic–pituitary–ovarian axis, capturing both physiological and pathological menstrual cycle patterns. Although the framework is not a fully mechanistic molecular model, it embeds key physiological feedbacks (e.g., estradiol–LH positive feedback, estradiol suppression of FSH, GnRH modulation of gonadotropins, LH-driven androgen production) that preserve functional relationships between hormones. By combining literature-derived parameters with stochastic interindividual variability, the model generates biologically coherent, temporally resolved hormone trajectories that can be exploited for unsupervised phenotyping, synthetic data generation for AI training, and educational applications. This positions the study within computational systems biology by providing a reproducible, simulation-based testbed linking biological knowledge with digital health tools.

Beyond the proof-of-concept presented here, the proposed framework is inherently extensible. Its parametric structure allows the incorporation of additional hormonal or metabolic markers, integration of more detailed mechanistic feedback loops, and calibration against empirical datasets as they become available. This flexibility means that the current implementation should not be viewed solely as a static data generator, but rather as the foundation of a modular simulation environment. Such an environment could support a broad spectrum of applications, from hypothesis testing in reproductive endocrinology to the development and benchmarking of AI-based diagnostic tools. By progressively expanding its biological realism and linking it to real-world measurements, this framework has the potential to evolve into a robust platform for in silico experimentation and personalized medicine approaches in women’s health.

### 4.1 Model fidelity and pathophysiological realism

The simulated hormonal profiles align well with known physiologic and pathologic signatures. Eumenorrheic individuals showed mid-cycle estradiol peaks and LH surges typical of ovulatory cycles [1], whereas PCOS profiles reproduced hallmark features such as chronic LH elevation, hyperandrogenism, and suppressed FSH, a pattern consistent with disrupted folliculogenesis and increased GnRH pulse frequency [6], [39]. The elevated AMH levels simulated in PCOS individuals also reflect clinical findings associated with persistent small antral follicles [40].

Importantly, the model reproduces dynamical hormonal behavior, not just point estimates. This is critical because hormonal dysregulation in PCOS is temporal as well as quantitative. Most prior models are static or based on averaged parameters [20], [31], [41]. Here, the use of high-resolution daily simulations represents a methodological advance, closer to real-life endocrine patterns.

### 4.2 Unsupervised phenotype separation: potential for clinical translation

The unsupervised clustering results demonstrate that simulated hormonal profiles alone, without diagnostic labels, can clearly separate PCOS from ovulatory phenotypes. This supports the potential utility of the model in phenotypic discovery, automated classification, and machine learning applications. Recent studies in reproductive medicine have explored similar unsupervised approaches using wearable biosensor data and biochemical inputs to predict ovulation or diagnose cycle abnormalities [42], [43].

Our findings suggest that AI tools trained on such synthetic hormonal data could aid in clinical scenarios where longitudinal data is incomplete or expensive to collect. By generating thousands of plausible endocrine trajectories, this framework could be leveraged to train classifiers or stratify patients in clinical decision support systems [44].

### 4.3 Clinical and technological applications

The present simulation model opens the door to a wide range of clinical and technological applications within reproductive endocrinology and digital health. It provides a dynamic visualization of hormonal rhythms that can be used in medical education, helping students, trainees, and even patients better understand the temporal patterns and inter-hormonal relationships across the menstrual cycle [45]. The contrast between eumenorrheic and PCOS profiles also supports training in diagnostic reasoning [46].

The model can be employed as a source of synthetic data to train machine learning algorithms, particularly in scenarios where real-world data is scarce, incomplete, or difficult to acquire. For example, virtual hormonal profiles may improve ovulation prediction algorithms, support phenotyping efforts, or enhance predictive tools for response to ovulation induction [21], [44]. Synthetic data generation also helps address privacy concerns while preserving biological realism for model development.

This work can contribute to the development of digital twins in reproductive health. A digital twin is a dynamic computational model of an individual that can be used to simulate and anticipate clinical outcomes. In fertility medicine, digital twins could help tailor stimulation protocols, forecast hormonal responses, or model treatment outcomes before actual intervention. Several recent frameworks have proposed integrating mechanistic models with data-driven personalization to improve decision-making in assisted reproductive technology (ART) [22].

Then, the model can provide a foundation for in silico trials, where thousands of virtual patients could be simulated to test hypothetical interventions (e.g., clomiphene, metformin, FSH injections) or to evaluate different PCOS phenotypes' responses [18], [47]. These simulations may complement or reduce the need for early-phase human studies, offering a cost-effective and ethical tool to optimize trial design and stratification.

Beyond visualization, the model serves as a generator of physiologically consistent, high-frequency synthetic data. Such datasets can be used to train and validate machine learning algorithms for cycle phase detection, ovulatory prediction, and phenotype classification, in contexts where large, curated longitudinal hormone datasets are not available. The same framework can support unsupervised phenotyping, early-stage digital twin prototypes, and educational tools for medical trainees and patients.

To facilitate adoption by researchers and clinicians, we are currently developing a user-friendly web interface based on the present framework. This platform will allow the generation of customizable multi-hormone synthetic datasets by specifying parameters such as phenotype, cycle length, and inter/intra-individual variability. Once completed, this tool will be made freely available to support applications in teaching, algorithm development, and simulation-based hypothesis testing.

Limitations

While our simulation framework provides biologically plausible endocrine profiles aligned with known physiological principles, its translational utility depends on empirical validation against real-world hormonal data. At present, no suitable multi-hormone longitudinal dataset was available to directly calibrate or validate the model. Consequently, this study focuses on the generation of physiologically plausible synthetic data as a proof-of-concept framework, with empirical integration planned in future work. A limitation of the present work is the absence of direct empirical validation against multi-hormone, multi-cycle datasets. While parameter choices were based on published physiological ranges, future work will integrate longitudinal clinical datasets to calibrate the model and quantitatively assess its performance in real-world settings. Future work should compare simulated trajectories with longitudinal hormone measurements collected from prospective cohorts using daily serum or urinary assays (e.g., estradiol, LH, FSH) across natural cycles. Studies such as Santoro et al. (2017) [1] and Usala et al. (2024) [20] have demonstrated the feasibility of high-frequency hormonal sampling in mid-reproductive and perimenopausal women, providing robust empirical baselines for comparison [1], [2]. Quantitative validation should include correlation coefficients (Pearson’s r), root mean square error (RMSE), and dynamic time warping (DTW) distance to assess the alignment of temporal patterns. Furthermore, statistical metrics such as coverage probability or prediction intervals could help quantify the fidelity of simulated hormone fluctuations relative to observed clinical distribution [8], [48]. Multi-cycle real-world datasets, such as those collected via urinary monitors (e.g., Mira™, Clearblue®) or wearable biosensors [42], would offer particularly valuable testbeds for validating both the amplitude and phase structure of hormone dynamics across individuals. Ultimately, integrating empirical data into a hybrid calibration framework, combining mechanistic constraints and statistical fitting, will be critical to transform this simulation model from a theoretical construct into a clinical-grade digital twin.

Moreover, the model presents other limitations. It is based on theoretical formulations and literature-derived averages rather than real-world longitudinal hormone datasets. Although interindividual variability was introduced stochastically, this cannot substitute for empirical calibration using patient-level data, particularly to capture rare phenotypes, non-linear transitions, or stochastic hormonal events (e.g., luteinized unruptured follicles). The hormonal curves are generated under fixed-length cycles (28 days), which does not reflect the natural variation in cycle length observed across populations, particularly in PCOS or perimenopausal women. Future versions should incorporate dynamic cycle length modeling based on probabilistic or individualized ovulatory events. The clustering and PCA analyses assume that mean hormone levels are sufficient for phenotype separation. This does not consider temporal dynamics such as peak timing, rate of change, or hormone interactions, features increasingly shown to carry diagnostic and predictive value. Then, while we modeled PCOS-like phenotypes, the heterogeneity of PCOS (e.g., NIH, Rotterdam, AES subtypes) is not yet represented. Subphenotyping PCOS within the model, and simulating comorbidities such as insulin resistance, would enhance its translational potential.

Although some feedback mechanisms between hormones are represented, they remain simplified and do not capture the full set of dynamic couplings of the HPO axis. The absence of fully coupled mechanistic equations currently limits the model’s use for causal simulation of interventions. Moreover, interindividual variability was introduced stochastically and does not substitute for empirical calibration using patient-level data, particularly to capture rare phenotypes or stochastic hormonal events. Future versions should incorporate dynamic cycle length modeling, probabilistic ovulatory events, and expanded phenotypic heterogeneity (e.g., PCOS subtypes with comorbidities). Although larger synthetic cohorts could be generated without technical limitation, we selected a population size consistent with prior simulation studies to balance representativeness and computational tractability. Future work will include formal sensitivity analyses to determine the minimal dataset size required for robust classification and phenotyping.

## 5 Conclusion

This study presents a dynamic, literature-based simulation of daily hormone fluctuations across the menstrual cycle in both eumenorrheic and PCOS-like virtual individuals. By embedding physiological principles and interindividual variability into a computational framework, we generated realistic endocrine trajectories that reflect known ovulatory and anovulatory patterns.

The model supports exploratory simulations, unsupervised classification of phenotypes, and generation of synthetic datasets for machine learning. While currently theoretical, this simulation constitutes a first step toward building personalized reproductive models and in silico tools for stratifying cycle disorders or predicting treatment responses. Integration with real-world data and clinical outcomes will be the key next step in transforming this framework from a conceptual simulation to a precision decision-support tool in reproductive medicine.

## Funding

This research received no external funding.

### Data sharing statement

Generated dataset is shown in supplementary file. Data access, responsibility, and analysis: Alexandre Vallée had full access to all the data in the study and takes responsibility for the integrity of the data and the accuracy of the data analysis.

## CRediT authorship contribution statement

**Jean-Marc Ayoubi:** Writing – review & editing. **Gaby Moawad:** Writing – review & editing. **Vallee Alexandre:** Writing - original draft, Conceptualization, Methodology, Simulation, Data analysis, Validation. **Anis Feki:** Writing – review & editing.

## Declaration of Competing Interest

The authors declare having no competing interests with this work.
